# Context dependency in risky decision making: Is there a description-experience gap?

**DOI:** 10.1371/journal.pone.0245969

**Published:** 2021-02-11

**Authors:** Inkyung Park, Paul D. Windschitl, Andrew R. Smith, Shanon Rule, Aaron M. Scherer, Jillian O. Stuart

**Affiliations:** 1 Department of Psychological and Brain Sciences, University of Iowa, Iowa City, Iowa, United States of America; 2 Department of Psychology, Appalachian State University, Boone, North Carolina, United States of America; 3 Department of Internal Medicine, University of Iowa, Iowa City, Iowa, United States of America; 4 Department of Psychology, Virginia Military Institute, Lexington, Virginia, United States of America; Texas A&M University, UNITED STATES

## Abstract

When making decisions involving risk, people may learn about the risk from descriptions or from experience. The description-experience gap refers to the difference in decision patterns driven by this discrepancy in learning format. Across two experiments, we investigated whether learning from description versus experience differentially affects the direction and the magnitude of a context effect in risky decision making. In Study 1 and 2, a computerized game called the Decisions about Risk Task (DART) was used to measure people’s risk-taking tendencies toward hazard stimuli that exploded probabilistically. The rate at which a context hazard caused harm was manipulated, while the rate at which a focal hazard caused harm was held constant. The format by which this information was learned was also manipulated; it was learned primarily by experience or by description. The results revealed that participants’ behavior toward the focal hazard varied depending on what they had learned about the context hazard. Specifically, there were contrast effects in which participants were more likely to choose a risky behavior toward the focal hazard when the harm rate posed by the context hazard was high rather than low. Critically, these contrast effects were of similar strength irrespective of whether the risk information was learned from experience or description. Participants’ verbal assessments of risk likelihood also showed contrast effects, irrespective of learning format. Although risk information about a context hazard in DART does nothing to affect the objective expected value of risky versus safe behaviors toward focal hazards, it did affect participants’ perceptions and behaviors—regardless of whether the information was learned from description or experience. Our findings suggest that context has a broad-based role in how people assess and make decisions about hazards.

## Introduction

When making decisions involving risk, one may learn about the potential risk in two different ways—from description or from experience. *Decision from description* (DFD) is a term that refers to when people make decisions after receiving unequivocal information about the values and likelihoods of possible outcomes, usually expressed numerically. *Decision from experience* (DFE) refers to when people make decisions without this unequivocal information. Instead, through repeated encounters, they must observe or experience how decision options play out in order to gain knowledge of possible outcome values and likelihoods. The term *description-experience gap* indicates systematic discrepancies between decisions made under these two formats or ways of learning about risk information [1–3].

Research on understanding description-experience gaps has proliferated over the past two decades. Most of the work has focused on gaps in people’s decisional tendencies relevant to rare events, but other types of gaps have also been addressed. For instance, recent studies have found that gaps may emerge in loss aversion [4,5], preference reversal [6], and ambiguity aversion [7–9]. Despite this growing literature on description-experience gaps, the question of whether a gap would also emerge in how context information influences risky decisions remains relatively unexplored. Here, we explore a possible description-experience gap in such context effects.

By *context effects*, we refer to situations where reactions to risk information about one hazard are affected by salient risk information about another hazard, even though the latter is not objectively relevant to the task at hand. These context effects can emerge in either one of two directions, *assimilation* or *contrast*. Consider a case in which people learn about a hazard, which we will call the focal hazard, that has a 20% likelihood of causing harm. In addition, they learn about another hazard, which we will call a context hazard, that has a 30% likelihood of causing harm. How will people respond when later faced with the focal hazard? The term *assimilation* refers to a shift in evaluated riskiness of the focal hazard towards the riskiness of the context hazard, whereas the term *contrast* refers to a shift in evaluated riskiness of the focal hazard away from the riskiness of the context hazard. In this example where the objective likelihoods are 20% for the focal hazard and 30% for the context hazard, an *assimilation effect* would indicate that the risk information about the context hazard makes the focal hazard seem more dangerous than it otherwise would. A *contrast effect* would indicate that the risk information about the context hazard would make the focal hazard seem less dangerous than it otherwise would.

A variety of studies using focal and contextual risk information have revealed contrast effects in people’s risky decision making [10–12]. However, previous studies relied on experimental paradigms that mainly involved DFD, and therefore it is unclear if a similar contrast effect would be observed in DFE. For this reason, a systematic comparison of DFD vs. DFE in context-dependent risky decision making is required. To determine if there is a description-experience gap in the degree to which context influences risk perception and decisions, we implemented a computerized game called the Decisions about Risk Task (DART).

Below, we review the current literature on description-experience gaps. We then provide a brief overview of the current DART paradigm and our rationale for determining if context effects differ across DFD and DFE with regard to the theoretical perspectives on context effects. Finally, we report the findings from two experiments that tested if there is a description-experience gap in how context affects risk perception and decision making.

### Description-experience gaps

Traditionally, studies on risky decisions were mostly restricted to DFD, using an approach often referred to as a gambling paradigm. In the typical gambling paradigm, individuals make a series of decisions among options that explicitly identify possible monetary outcomes and their probabilities in a numerical format. However, the paradigm does not fully capture the range of behaviors that are evident in our everyday decisions; we often make choices based on past experiences without having a clear outline of the outcomes and probabilities related to the risk. Consequently, the inclusion of DFE in this research has been growing over the last few years [1,2,13,14]. Many DFE paradigms build on classical gambling paradigms, whereby participants are prompted to choose a gamble of their preference. However, in DFE paradigms, participants are not provided with known probabilities nor outcomes associated with gambles. Instead, they obtain information regarding the distribution of a given gamble by either actively making or passively watching iterative choices and the resultant outcomes [3,15].

By implementing DFE paradigms, researchers have looked for description-experience gaps in diverse decision phenomena. The most well-known description-experience gap involves decisions regarding probabilistically rare events, conventionally defined as events with less than a 20% chance of occurring [2,3,15]. In DFD paradigms, individuals show decision patterns in which they are overly sensitive to the possibility of a rare event. However, in DFE paradigms, they show decision patterns in which they are less or even under-sensitive to the possibility of a rare event [3]. This particular form of a description-experience gap has seen a great deal of attention recently. In fact, the term “description-experience gap” is often interpreted as necessarily referring to this particular type of gap. However, it is important to note that other forms of description-experience gaps have been investigated. As noted earlier, such gaps have been found in the context of loss aversion [4,5], preference reversal [6], and ambiguity aversion [7–9].

In particular, the type of description-experience gaps most relevant to the current study are those of the decoy effect [16–18]. The decoy effect—also known as the attraction effect or the asymmetric dominance effect—refers to a change in preference among equally compelling options when a less-attractive ‘decoy’ alternative is added to the decision context [19]. Specifically, the choice share of the option that is similar to, but dominates, the decoy, increases.

Recent findings suggest the presence of a description-experience gap in the decoy effect [16–18]. Ert & Lejarraga [16] studied the decoy effect in choices among gambles. In their studies, the decoy effect was observed in DFD, but not in DFE. It is important to note that the decoy effects in DFE were absent mainly because it was harder for participants to identify the dominated status of a decoy option when the option sets were learned from experience rather than from description [16–18]. Similarly, Hadar et al. [18] also demonstrated the description-experience gap in the decoy effect, but when the differences between the options were made prominent and easier to identify, participants who were able to recognize the dominance relationship among the options exhibited a decoy effect in DFE. Participants who failed to recognize the dominance relationship did not exhibit a decoy effect. To summarize, the current literature suggests that the presence of a decoy may differently affect choices among gambles depending on whether the choice options were learned from experience vs. description, but only to the extent that people fail to identify and distinguish a key difference between the options learned from experience.

Although research on the decoy effect provides some evidence about how description-experience gaps might or might not be relevant to context effects, the decoy effect is one specific example within a broad range of phenomena influenced by context. We aimed to investigate the influence of the DFD vs. DFE formats on the directionality and the magnitude of a basic form of a context effect—namely, how the probabilistic risk level of one hazard would influence decisions about another risky hazard. Our approach to studying context effects shares some broad features with the studies on decoy effects in choices among gambles, but our operationalizations and paradigm are distinct. Our project was not designed as a specific extension of findings on the decoy effect, but it does speak to whether those findings about description-experience gaps generalize across different forms of context effects.

### Overview of the decisions about risk task

We used a specific task—the Decisions about Risk Task (DART)—to study this context effect. The DART was originally developed to instantiate a virtual experimental environment where people learn about risks/hazards and then make decisions in light of those risks/hazards [20]. In the current version of the DART, participants played a virtual character with the goal of accumulating as many points as possible throughout the task. Participants first learned about how often two hazards they would encounter might explode. Then they had to make a series of risky decisions related to those hazards.

Critically, two between-participant manipulations were used to test for a description-experience gap in context effects. First, we manipulated the *learning format* in which participants acquired likelihood information about two hazards. Some participants were given numerical descriptions about the explosion rates of the hazards (i.e., DFD format), whereas other participants could only learn about the likelihood information from a series of demonstration trials from which they could estimate the explosion rates of the hazards (i.e., DFE format). Second, the *context risk rate* was manipulated. The explosion rate of one of the hazards (hereafter called the *focal hazard*) was always fixed at same level (i.e., at 33%), whereas the explosion rate of the second hazard (hereafter called the *context hazard*) was varied across groups (i.e., at 20% or 46.7%).

After they learned about the risk information, participants encountered a series of test trials. In each trial, they needed to choose between a safe path and a risky path (i.e., a path on which a hazard’s explosion would cause harm). Taking the risky pathway could result in either points gained from retrieving a coin or points lost if a hazard exploded. Meanwhile, the safe pathway always offered neither a gain nor loss of points.

On each trial, we recorded the participants’ decisions, allowing us to calculate the proportion of risky choices per hazard by conditions. Our key interests were 1) how the proportion of risky choices for the *focal hazard* varied as function of the context risk rate, and 2) whether the direction or magnitude of this effect varied as a function of learning format. Our study also included measures of the perceived likelihood of the hazards exploding.

### Predictions and theoretical approaches for contrast vs. assimilation in context effects

We began this study with a clear prediction about how the context-risk-rate manipulation would influence behavior toward the focal hazard in a DFD format. Specifically, we expected to observe contrast effects. The grounds for this prediction come from previous evidence that when people interpret the meaning of numeric risk information, they often compare that information to other salient risk information, thereby causing contrast effects [10–12,21]. For example, Windschitl et al. [11] reported that even when participants in two groups were both informed that women had a 12% risk for a target disease, they held different intuitive beliefs about how vulnerable women were, as a consequence of one group being told that men’s risk of the same disease was 4%, and the other being told that it was 20%.

There are several theoretical perspectives that explain contrast effects in DFD formats. One group of accounts posits that a context stimulus or value can be used as a comparison standard and can shift the evaluation or categorization of the target. When the focal stimulus or value is distinct and sufficiently different from a contextually salient stimulus or value, the context serves as a comparison standard (causing contrast) rather than as informational context that could have an assimilative influence [22,23]. A closely related notion is that the contextual stimulus changes the categorization of a focal stimulus. In the present study, the focal hazard might be categorized as the “more” or “less” dangerous one as a function of the explosion rate of the contextual hazard. This categorization itself, which might have gist-like properties, may impact the response to the focal hazard, even when one is aware of the more precise and verbatim rate at which the focal hazard causes harm [24–27].

A selective accessibility account proposed by Mussweiler [28] posits that the directional influence of context information is shaped by the initial holistic assessment of focal-context similarity or dissimilarity. This holistic assessment influences individuals to generate different hypotheses that guide further information search and interpretation. Assuming that an explicitly stated hazard-explosion rate of 33% is immediately viewed as different from a rate of 20% or 46.7%, this immediate assessment might shape expectations and what is noticed about the focal hazard that was said to have the 33% rate.

Range-frequency theory [29] assumes that evaluations of virtually any stimuli, even explicit cardinal quantities, are shaped by contextual stimuli. This theory highlights the influence of both the range and the frequency distribution of contextual stimuli. Applied to the present study, one could argue that the subjective riskiness of a focal hazard that explodes at 33% is evaluated differently according to how the context rates shape where 33% falls in the range of possible rates, and where 33% falls within the rank order of possible rates.

Lastly, a theory called decision by sampling [30] shares some similarities with range-frequency theory in how it accounts for contrast effects. According to decision-by-sampling, evaluating a particular attribute of a focal stimulus involves pairwise comparisons within the sample distribution of that attribute. That is, the focal attribute is compared in terms of its relative rank among other attributes that are mainly sampled from the memories of past encounters or external contexts that are mentally available to the decision-maker. Following this logic, the model predicts a contrast effect because the presence of a salient context rate would influence the result tallies of the pairwise comparisons used to drive decisions about the focal hazard.

In summary, there are a number of theoretical perspectives that attempt to explain and predict contrast effects in people’s evaluations and responses to a focal hazard when risks are communicated explicitly in a numerical format (i.e., in DFD). With this in mind, a key goal of the current study was to compare how context would influence decisions in DFE vs. DFD. To evaluate whether the specific theoretical perspectives mentioned above predict differences in context effects across DFE vs. DFD, we must first appreciate the following fundamental difference between DFE vs. DFD: unlike DFD, learning about the risk rates in DFE is an incremental process. It requires additional cognitive operations, such as encoding outcomes of experienced events and updating previously held beliefs about a given hazard’s riskiness. As such, when a person is early in the process of learning risk rates in DFE, there would be no means by which the person could confidently judge the relative risk rates from two hazards. Only after numerous trials (or iterative learning opportunities) could they make a confident judgment about how often each hazard causes harm, whether the threat levels are approximately the same or different, and, if different, which hazard holds the greater threat.

Given the *incremental* learning involved in the DFE format, some theoretical perspectives would predict reduced or absent contrast effects in DFE. Specifically, some perspectives would suggest that if differences between focal and context stimuli cannot be initially detected, robust contrast effects will not be observed. For instance, if we apply a selective accessibility account [28] in DFD, the two hazards are known to be different in their explosion rates—triggering further “difference” expectations and biased processing among most or all participants. However, in DFE formats, there is no immediate sense of the overall difference or similarity. Given noise in the early experienced trials, many participants might initially think of the two hazards as exploding at similar rates, or they might even have an inverted impression of the direction of difference in the explosion rates. Furthermore, some perspectives would lead to the assumption that if there is ambiguity in the evaluation of focal and context rates—as there would be in DFE formats—this might lower the chances that a context rate would be used as an evaluation standard, or that it might not trigger the two rates to be categorized differently [31–33]. This would reduce any contrast effect, if not promote assimilation.

There is another potential source of bias that we have not yet mentioned that is relevant to only DFE. It offers another reason why context effects might have different strength or direction in DFE relative to DFD. Namely, in DFE, error prone memory may lead to failure in distinguishing or inhibiting the irrelevant and independent context events when assessing the riskiness of focal events. This may ultimately bias the perceived rate of the focal risk [34,35]. One may incorrectly recall the explosion of the context hazard as an event related to the focal hazard or vice versa, blurring the distinction between explosion rates related to two different types of hazards. This blurring would essentially yield a pattern of results that fits the assimilation direction. For example, when a context hazard has a higher explosion rate compared to the focal hazard, the focal hazard may also be perceived to have a similarly high explosion rate—higher than it otherwise would. Again, this source confusion only happens in DFE, and it therefore provides a potential reason why context effects might have a different strength or direction in DFE relative to DFD.

Not all of the accounts described assume different results for DFE and DFD. Specifically, both decision-by-sampling and range-frequency theory suggest that information acquired through experience will generate an internal distribution of the attribute (i.e., the explosion rate), facilitating ordinal comparisons between the focal and context hazard, and thus a contrast effect. Specifically, decision-by-sampling suggests that the judgments about the focal attribute are made by comparing the relative rank of the value of the focal attribute to an attribute value selected from the context distribution. Similarly, range-frequency theory emphasizes that the focal and the context attributes are compared in terms of frequency and range of the possible values. Both accounts acknowledge that the distribution of context attributes can emerge not only from descriptive information about the relevant event, but also from a series of encounters with the event. Following this logic, these models predict contrast effects regardless of the learning format.

Recent findings using neurophysiological approaches are also consistent with an expectation of contrasts effect in DFE [36–38]. That work suggests that the value of an option is encoded relatively rather than absolutely. For instance, in Palminteri et al. [36], participants were shown choice pairs that either held overall positive or negative expected values. When participants succeeded in avoiding a loss in a choice set with an overall negative expected value, such behavior was reinforced similar to when receiving a reward in a choice set with overall positive expected values. This relativity in valuation is reflected at the neural level, whereby encoding of a negative outcome could engage brain areas involved in either reward vs. punishment processing, depending on the context.

In summary, we predicted a robust contrast effect in DFD. However, the existing literature on DFE is equivocal. Categorization accounts and the selective accessibility account predict that there will be an attenuated contrast effect, or even an assimilation effect in DFE. Furthermore, the source confusion account specifies mechanisms that might be in play in DFE that would bias responses in an assimilative, rather than contrastive, direction. Meanwhile, decision-by-sampling and range-frequency theory, as well as recent findings from literature using the neurophysiological approach, point to contrast effects in DFE as in DFD.

### Overview of the studies

To investigate the effect of learning format on the direction and magnitude of the context effect in risky decision making, we conducted two studies. The studies used the DART paradigm, which instantiates a virtual environment that allows people to learn about probability information (i.e., hazard risk rates) and outcome information (i.e., coin related points) and make decisions based on the acquired information. In both studies, we examined how decisions about the focal hazards were influenced by manipulations of context-risk rates and learning formats. For the learning-format manipulation in Study 1, some participants had to learn risk rates only incrementally from iterative experiences (i.e., experience condition), whereas other participants were given a summary description of the risk rates after also having iterative experiences (i.e., description condition). In Study 2, which was largely a replication of Study 1, this learning-format manipulation was made a bit purer—with iterative experiences being completely removed from the learning phase within the description condition. Studies 1 and 2 also differed somewhat in how likelihood information was solicited. Finally, in both studies, the values of coins that could be gained by making a risky decision were varied per trial. While the impact of coins is not a main focus of this paper (but is briefly reported), the variation in coin values kept the task interesting and challenging to participants, because it varied the expected values for risky vs. safe options in the task.

## Study 1

### Methods

#### Participants and design

The participants (N = 303) were students from introductory psychology courses at the University of Iowa. Participants provided verbal informed consents after reading the consent form approved by the University of Iowa Institutional Review Board and they were recorded via data entry. The written consent was waived by the board. The design was a 2 (Learning Format: Experience vs. Description) x 2 (Context Risk Rate: 20% vs. 46.7%) x 3 (Coin Amount: 70, 85, or 100) mixed factorial. The first two factors were between-participant manipulations.

The target sample size was set at 300. Although the target size was set *a priori*, it was not based on a formal power analysis. Instead, it was a rough estimate of what would provide reasonable power, shaped by our experience with other studies using the DART paradigm. The final sample size of 303 allowed for 80% power to detect a medium sized contrast effect (*d* = 0.45) within a given learning format condition or to detect a small-to-medium sized interaction (*f* = 0.16) between learning format and context risk rate [39].

#### Procedure and task

Participants were tested at individual computers. After a consent process, participants were introduced to DART. The introduction included the following information.

**Fig 1:**
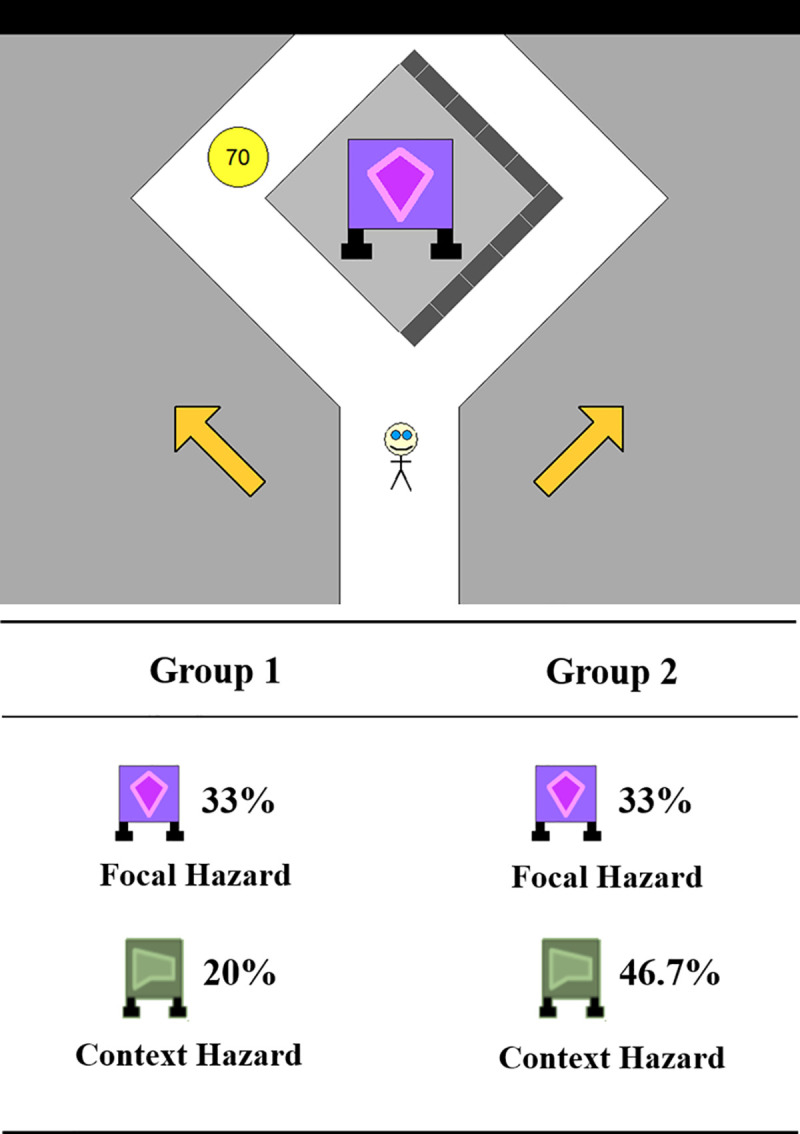
Decisions about Risk Task (DART). (A) Illustration of a trial in the DART used in Study 1 and 2. (B) Illustration of between-participant context risk rate manipulation. Focal hazards had identical risk rate across groups while context hazards differed across groups.

1. Participants would be playing a virtual game in which their goal was to earn as many points as possible before reaching the end of a journey. If they earned more points than the average player, they would win a candy bar.
2. Participants would control an avatar representing themselves that traveled along a path, and there were opportunities to collect coins that were worth varying amounts of points.
3. In a given trial, the avatar would encounter a crucial point where the path split in two, and in the middle of the two pathways there would be an “abandoned device” called a *gurg*. A gurg would either crumble or explode when the avatar traveled past it.
4. Participants had to decide which side of the split the avatar should travel on. They were told that one side of the split would be lined with a wall that protected an avatar from getting caught in a possible gurg explosion (i.e., safe pathway) while the other side would have no protective wall, but it would have a coin worth a specified amount of points (i.e., risky pathway). More specifically, participants were instructed that if they choose to travel along an unprotected pathway, they would have the opportunity to collect coin that is worth either 70, 85, or 100 points. However, they would leave themselves open to damage from a potential explosion. They were also told that if they are damaged by an exploding gurg, they would lose 250 points.
5. Participants would have to make a decision by the time their avatar reached the split (5 seconds), otherwise they would lose 20 points repeatedly until the choice was made.
6. There were two types of gurgs that could be encountered, which had different tendencies regarding how often they would crumble or explode. The overall layout of a given trial is provided in Fig 1A.

At this point, the instructions provided more information on how the participants would learn about the tendencies of the two gurgs. In the *experience group*, participants read: “In preparation for the journey, you will now watch several trials in which these devices crumble or explode. By watching what a particular type of device tends to do (i.e., crumble or explode), you learn useful information about what that type of device will do on the journey.” They then passively viewed 50 automated demonstration trials played back-to-back. In a given demonstration trial, participants would see the avatar passing by a gurg that either exploded or harmlessly crumbled. Overall, the rates of exploding vs. crumbling in the demonstration trials were proportional to the actual explosion rates of the hazards in the main trials. Both types of gurgs were presented an equal number of times across 50 trials.

Participants in the *description group* were also introduced to the same 50 demonstration trials. However, upon the completion of the demonstration trials, they were given additional information—namely explicit, numeric information about the explosion rate of each hazard (e.g., “The explosion rate for the Orange gurg is 20%. In other words, when the Orange gurg is shown, it will explode 20% of the time.”).

Crucially, the explosion rate for one of the two gurgs was manipulated between participants. For half the participants (Group 1 in Fig 1B), the rates for the two gurgs were 33% and 20%. For the other participants (Group 2 in Fig 1B), the rates were 33% and 46.7%. We call the gurg that exploded at a 33% rate the focal gurg. We call the other gurg the context gurg. Note that in the perspective of participants, the two gurgs would appear to differ only in explosion rates, and that the distinction between the focal and context gurgs would not be known to them.

After learning about the explosion rates of the gurgs, and before the start of the main test phase of the DART, participants were informed about mini-games that would be included between test trials (one mini-game per interlude). The mini-games were very simple, brief, and conceptually unrelated to the purpose of the DART and to the test trials described below (e.g., using the computer mouse to guide one’s avatar on the chase of a floating coin). They were inserted to simply break up what otherwise may have been a monotonous series of trials.

The test phase contained 60 trials (Fig 1A). Each trial proceeded in a manner consistent with the introductory instructions seen by the participants. The avatar approached the split at a fixed speed. Participants could see where the coin and protective wall were located, and what coin amount was offered in that trial. To select their path at the split, participants would click on one of the arrows that pointed toward the left and right pathways. If the response time exceeded 5 seconds, the avatar would bounce against the split and a “-20” would drift out from that area to signify the loss of 20 points. Once participants indicated their decision, the feedback was provided in a form of animation displaying the gurg either exploding or crumbling, with the points lost or earned shown on the side, respectively. In case of a gurg explosion, participants who chose the risky pathway kept the points from the coin but also suffered loss of 250 points. The total points that participants had accrued throughout the task were shown at the corner of the screen.

Total of 60 trials were divided into two blocks with 30 trials each. On a given trial in the test phase, participants would encounter one of the two gurgs and a coin worth one of three amounts (70, 85, or 100 points). The coin values were independent of explosion rate. Consequently, a given participant experienced repetitions of six unique trial types. Table 1 provides the summary of the six trial types and their expected value. The six trial types were repeated ten times respectively. These repetitions were block randomized such that within the first block, all six trial types happened five times. On all trials, the coin appeared opposite the protective wall, and the relative positions of the safe and risky paths were counterbalanced across trials.

**Table 1:** Summary of expected values (EV) offered in risky and safe choice in Study 1 and 2.

**Study 1**			Risky Pathway			Safe Pathway
			Points Lost (p)	Points Won (p)	EV	EV
	Focal	Identical across groups	-250 (0.33)	70 (1)	-12.5	0
			-250 (0.33)	85 (1)	2.5	0
			-250 (0.33)	100 (1)	17.5	0
	Context	20% Context group	-250 (0.20)	70 (1)	20	0
			-250 (0.20)	85 (1)	35	0
			-250 (0.20)	100 (1)	50	0
		46.7% Context group	-250 (0.46)	70 (1)	-45	0
			-250 (0.46)	85 (1)	-30	0
			-250 (0.46)	100 (1)	-15	0
**Study 2**			Risky Pathway			Safe Pathway
			Points Lost (p)	Points Won (p)	EV	EV
	Focal	Identical across groups	-150 (0.33)	50 (0.67)	-16	0
			-150 (0.33)	75 (0.67)	0.75	0
			-150 (0.33)	100 (0.67)	17.5	0
	Context	20% Context group	-150 (0.20)	50 (0.80)	10	0
			-150 (0.20)	75 (0.80)	30	0
			-150 (0.20)	100 (0.80)	50	0
		46.7% Context group	-150 (0.46)	50 (0.54)	-42	0
			-150 (0.46)	75 (0.54)	-28.5	0
			-150 (0.46)	100 (0.54)	-15	0

Points Lost/Won indicate the amount of points that could be won or lost by choosing the given risky option, and (p) indicates its probability. Safe choice guaranteed zero points at 100% probability.

Additionally, for twelve random trials within the second block, participants were asked to give a likelihood judgment upon the presentation of the trial. In those trials, participants had to indicate how likely it was that the gurg would explode. They responded with a slider scale marked every tenth percent that ranged from ‘0%, Definitely will not explode’ to ‘100%, Definitely will explode’. After they submitted the response, the trial resumed as normal, and participants submitted their choice between the risky or safe pathway.

After completing the DART, participants were asked to answer demographic questions and exploratory measures before being debriefed. Those who earned points above the average received a candy bar of their choice.

### Results

#### Preliminary notes

We analyzed the data from the trials involving the context gurg separately from the trials involving the focal gurg. Again, the explosion rate of the focal gurg was the same across all participants (33%), whereas the rate for the context gurg was 20% for half of the participants but 46.7% for the other half. Our primary interest was in decisions and likelihood judgments about the *focal gurg*. However, for each outcome measure, we start by describing the results for the context gurg. Descriptive statistics are provided in upper panel of Table 2.

**Table 2:** Summary of descriptive statistics for Studies 1 and 2.

**Study 1**	Context Hazard Risk Rate		% of Risky Choices		Likelihood Judgment	
			Experience	Description	Experience	Description
	Focal Trials	C = 20%	59.82 (18.9)	57.53 (17.16)	48.76 (14.2)	41.51 (11.62)
		C = 46.7%	64.68 (13.59)	68.92 (14.20)	51.42 (12.28)	42.74 (10.38)
	Context Trials	C = 20%	68.18 (19.08)	69.00 (15.99)	46.31 (15.55)	34.42 (14.94)
		C = 46.7%	56.58 (18.68)	54.01 (17.00)	55.85 (11.55)	48.93 (8.66)
**Study 2**	Context Hazard Risk Rate		% of Risky Choices		Likelihood Judgment	
			Experience	Description	Experience	Description
	Focal Trials	C = 20%	51.83 (21.73)	50.34 (22.60)	4.55 (0.89)	4.22 (0.97)
		C = 46.7%	56.13 (19.90)	56.57 (21.11)	4.30 (0.88)	3.96 (0.98)
	Context Trials	C = 20%	60.45 (20.26)	63.77 (20.84)	4.03 (0.89)	3.74 (1.08)
		C = 46.7%	42.90 (22.32)	42.86 (22.66)	4.72 (0.86)	4.65 (0.90)

Parenthetical numbers reflect standard deviations. C and F indicates risk rate of context and focal hazard, respectively. Note that focal risk rates (i.e., F = 33%) are identical across all context risk rate conditions. For the choice dependent variable, a contrast effect would be evident when the rate of risky choices on focal trials is higher in the group where the context-hazard risk rate is 46.7% than where it is 20%. For the likelihood-judgment dependent variable, a contrast effect would be evident when the judgments on focal trials is lower in the group where the context-hazard risk rate is 46.7% than where it is 20%.

Preliminary analyses revealed that the counterbalancing of gurg color across focal vs. context assignments and the position of the protective wall did not substantially change interpretation of any key results, so we have collapsed across the counterbalancing for all subsequent analyses. In the ANOVAs reported below, coin amount always had main effects in a sensible direction. That is, people were more likely to choose the risky path when the trial involved a high-value vs. low-value coin. Aside from a few minor exceptions, coin amount did not interact with other key factors. Given that our main hypotheses did not differ as a function of the coin amount, we will only briefly report on the effects of coin below.

For each set of ANOVAs described in the sections below, full statistical reporting can be found in the supporting information (S1-S4 Tables in S1 Appendix). Given our main dependent variables were proportions (per cell), we also checked if arcsine square root transformations of the proportion data would substantially influence any of our main results from the ANOVAs [40]. They did not. Therefore, we report the ANOVAs on the untransformed data. A logistic regression approach to analyzing the data yields the same conclusions (see S5 Table in S1 Appendix for the logistic regressions).

#### Decisions about context gurgs

**Fig 2:**
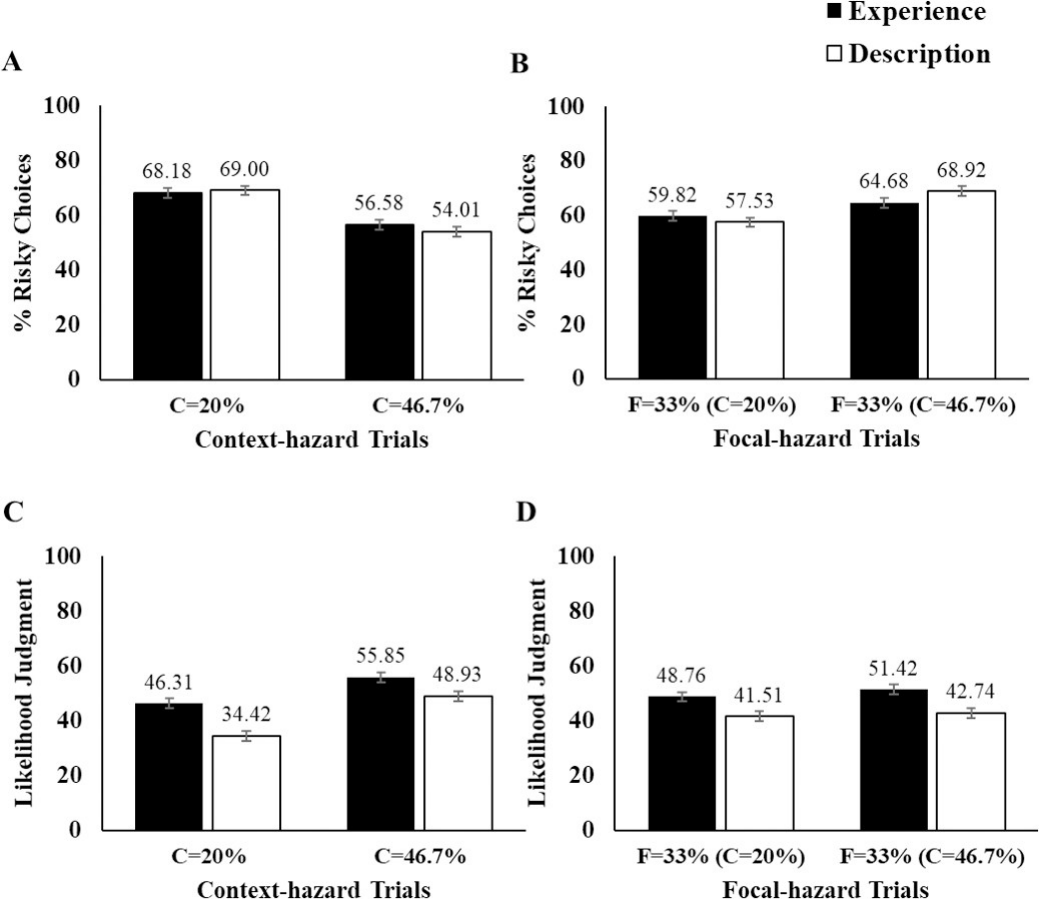
Summary of Study 1 results. C and F indicate risk rates of context and focal hazard, respectively. Note that focal risk rates (i.e., F = 33%) are identical across all context risk rate conditions. (A) Mean proportion of risky choices in context-hazard trials, (B) Mean proportion of risky choices in focal-hazard trials, (C) Mean likelihood judgment for context hazards, (D) Mean likelihood judgment for focal hazards. Black bars indicate the experience condition and white bars indicate the description condition. The numbers above the bars indicates the mean for each condition. Error bar indicates ± 1 S.E.

**Fig 3:**
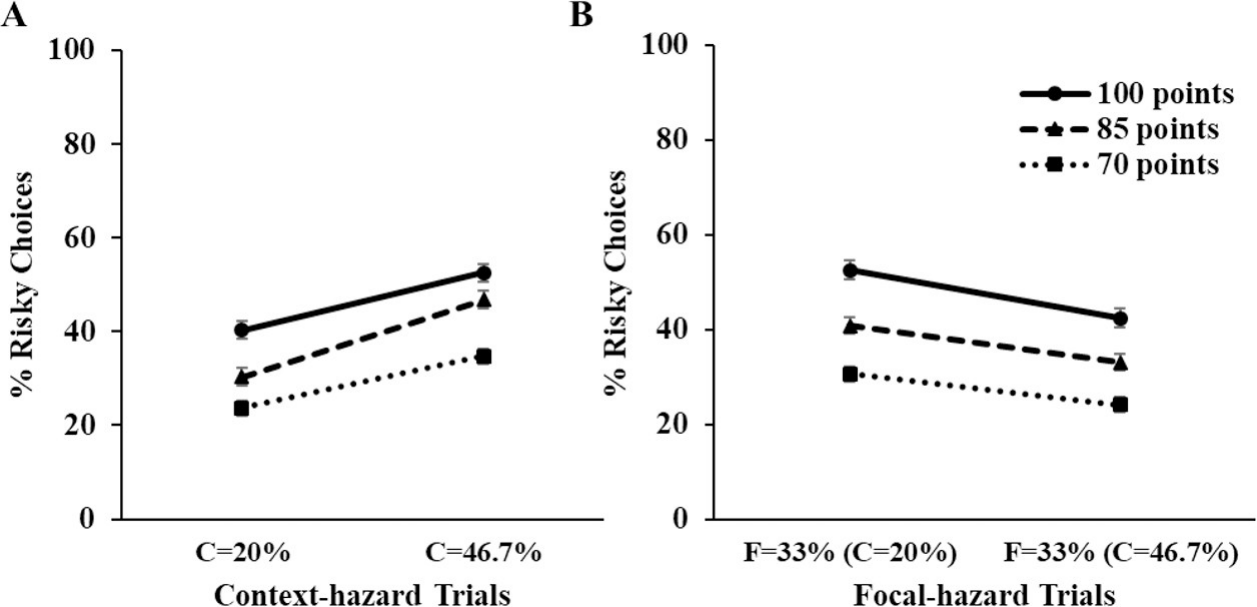
Summary of Study 1 results regarding coin manipulation. C and F indicate risk rate of context and focal hazard, respectively. Note that focal risk rates (i.e., F = 33%) are identical across all context risk rate conditions. (A) Mean proportion of risky choices in context-hazard trials across varying amount of coins, (B) Mean proportion of risky choices in focal-hazard trials across varying amount of coins. Error bar indicates ± 1 S.E.

For each combination of block and coin, we calculated the percentage of times (out of five) a participant chose the risky path for trials involving a context gurg. We submitted these values to a 2 (Block) x 3 (Coin) x 2 (Context Risk Rate) x 2 (Learning Format) ANOVA, with the first two factors as repeated measures (Fig 2A). This analysis may be considered as a manipulation check to make sure participants were sensitive to the relevant outcome and probability information on a given trial. The results revealed that participants were indeed sensitive to the information. A main effect of context risk rate showed that participants encountering a context gurg were more likely to take a risk when the explosion rate for the context gurg was 20% as opposed to 46.7%, *F*(1, 299) = 42.59, *p* < .001, *η*_*p*_^*2*^ = .13 [41]. Also, the main effect of coin revealed that participants were more likely to take risks as the amount of coin increased, *F*(1.81, 298) = 83.37, *p* < .001, *η*_*p*_^*2*^ = .22 (Fig 3A). These effects of explosion rate and coin were not significantly qualified by the format (experience or description), nor block. No other notable effects were significant (see S1 Table in S1 Appendix for full statistics).

#### Decisions about the focal gurgs

Our primary interest was in how the context risk rate would impact decisions made about the focal gurg—which always had the same explosion rate (33%)—and whether this impact would vary as a function of learning format. For each combination of block and coin, we calculated the percentage of times (out of five) a participant chose the risky path. We submitted these values to a 2 (Block) x 3 (Coin) x 2 (Context Risk Rate) x 2 (Learning Format) ANOVA (Fig 2B). As expected, coin amount again significantly affected risky choices; people made more risky choices when the larger coin amounts were offered, *F*(1.89, 298) = 94.02, *p* < .001, *adj* ${\hat{\eta }}_{p}^{2}$ = .24 (Fig 3B). More importantly, the main effect for context risk rate was also significant, *F*(1, 299) = 19.25, *p* < .001, *adj* ${\hat{\eta }}_{p}^{2}$ = .06. Participants were more likely to take risks in focal gurg trials when the context gurg exploded at a 46.7% rate than at a 20% rate. This pattern confirms a contrast effect; participants’ risk taking tendency in focal gurg trials (which maintained an identical explosion rate across the groups) was influenced by the explosion rate of the context hazard. Risk tasking on these focal trials was higher when the context risk rate was high rather than low. However, this pattern did not significantly differ as a function of learning format, *F*(1, 299) = .28, *p* = .598. The contrast effect was about the same among participants in the description group as it was among participants who only learned about the likelihood information incrementally from experiences (i.e., experience group). Given the potential importance of this null effect, we computed the Bayes factor for it (*BF*_*01*_ = 3.61). *BF*_*01*_ values at 1 are considered as no evidence, whereas *BF*_*01*_ values of 1 to 3 are considered as showing anecdotal evidence for the null hypothesis, 3 to 10 as substantial, 10 to 30 as strong, 30 to 100 as very strong, and values greater than 100 as decisive evidence against the alternative [42]. Therefore, the *BF*_*01*_ of 3.61 implies substantial evidence for the null.

The contrast effect did not significantly interact with the coin amount, but there was a small, significant interaction with the block, *F*(1, 299) = 5.45, *p* = .020, *adj* ${\hat{\eta }}_{p}^{2}$ = .02. Namely, we found significant contrast effects both in the first and the last block, and the magnitude of contrast effect was slightly larger in the first blocks than in the last.

#### Likelihood judgments about the context gurgs

For each level of coin amount, we calculated the average likelihood judgment from the trials involving a context gurg. We submitted these rates to a 3 (Coin) x 2 (Context Risk Rate) x 2 (Learning Format) ANOVA, with the coin factor as a repeated measure (Fig 2C). The main effect of coin was not significant, *F*(2, 298) = .84, *p* = .434. This result was not surprising considering that the likelihood judgment trials solicited the assessment of the explosion rate of the gurg independently from the outcome information such as the coin amount. The significant main effect of the explosion rate indicated a sensible pattern where participants assessed the context gurg with 46.7% explosion rate to be more likely to explode than the one with the 20% explosion rate, *F*(1, 299) = 64.88, *p* < .001, *adj* ${\hat{\eta }}_{p}^{2}$ = .18. Lastly, we also found a significant main effect of learning format, *F*(1, 299) = 39.68, *p* < .001, *adj* ${\hat{\eta }}_{p}^{2}$ =. 12. Specifically, overall likelihood judgments from the description group were lower than that of the experience group.

#### Likelihood judgments about the focal gurgs

As with likelihood judgments for the context gurg, we submitted likelihood judgments for the focal gurg to a 3 (Coin) x 2 (Context Risk Rate) x 2 (Learning Format) ANOVA, with the coin factor as a repeated measure (Fig 2D). The main effects for coin, *F*(2, 298) = .99, *p* = .371, and the explosion rate, *F*(1, 299) = 1.91, *p* = .167, were not significant. We found significant main effect in the learning format, *F*(1, 299) = 32.31, *p* < .001, *adj* ${\hat{\eta }}_{p}^{2}$ = .10. Again, participants in the description group had lower likelihood judgments compared to the experience group.

### Discussion

The main goal of Study 1 was to investigate the direction and the magnitude of influence that a context hazard exerts on the decisions about a focal hazard, and whether this influence is affected by the format by which people learn risk information. We found a significant effect of the context risk rate. Specifically, participants’ decisions regarding the focal gurg differed as a function of the context gurg. The pattern of responses indicated a contrast effect; participants made choices as if the riskiness of the focal gurg was in contrast to that of the context gurg. Participants in the condition where the context gurg’s explosion rate was at 20% made fewer risky decisions on focal-gurg trials than did participants in the condition where the context gurg’s explosion rate was at 46.7%. This contrast effect was not moderated as a function of the learning format—i.e., whether people learned about gurgs’ explosion rates only by observing the demonstration trials vs. by also receiving explicit, numeric rate information. Said differently, there was no description-experience gap. These results broadly favor the perspectives that predict contrast effects in both DFD and DFE (i.e., decision-by-sampling model and range-frequency theory) over the accounts that predict attenuated/null contrast effects in DFE (i.e., categorization accounts or selective accessibility account).

Alternatively, the lack of description-experience gap could be explained by a methodological issue in the current design. Namely, in the “description” condition, participants were given descriptive information about the hazards only after they had iterative experiences in the demonstration trials. A more complete name for this condition might be the “experience and description condition.” This methodological feature perhaps weakened the operationalized difference between our DFD and DFE manipulation. It is still instructive that the numerical information presented in current description condition did not seem to alter the observed contrast effect. However, these results leave open the question of whether the description-experience gap would be observed if the descriptive information in the description condition was presented immediately, rather than after experiential learning had started. This issue was addressed in Study 2 by refining the manipulation of learning format.

An unsurprising feature of the results from Study 1 was that coin amounts influenced choices. Participants made more risky choices when the coin amount on the risky path was larger rather than smaller. This indicates that the participants were, in general, capable of discerning advantageous vs. disadvantageous options based on the expected values. At the same time, these distinctions made between the advantageous vs. disadvantageous options were far from perfect; we did not observe fully-consistent risk-seeking behavior for advantageous options nor fully-consistent risk-averse behavior for disadvantageous options. This is understandable given the dynamic nature of the DART paradigm which does not easily allow participants to explicitly calculate the expected values offered in a given trial.

An additional issue is whether participants’ likelihood judgments showed patterns that matched those on the behavioral choice measures. They did not. First, there was a significant main effect of the learning format for both the context- and focal-gurg trials. That is, participants in the experience condition judged the gurgs more likely to explode overall, compared to those in the description condition. We speculate that this merely reflects the consequences of numeric anchoring. Participants in the description condition were given an explicit numeric risk rate, on which their later probability estimates tended to be anchored. There was no such anchor for participants in the experience condition. Without that anchor, and with uncertainty about the actual risk levels, they were more likely to use mid-level probabilities, which were overestimates.

Second, we did not find context effects in likelihood judgments as we did in risky choices. That is, participants’ likelihood judgments about the focal gurg did not differ as a function of the context gurg’s explosion rate. The possible reasons for such inconsistency could be related to when and how we solicited the likelihood judgments. The likelihood judgment trials were presented only in the second half of the test phase. As reported, the context effect on behavior measures was larger in the first block of trials than in the last block. Therefore, it is possible that participants’ impressions of likelihood were initially affected by context information, but this effect faded by the time we solicited them. Furthermore, we solicited likelihood judgments on numeric scales, which may have encouraged a response strategy that precludes sensitivity to the contextual effects [11,43–45]. For instance, when providing a numeric likelihood judgment for the focal hazard, participants may have deliberatively dwelled on proportion information they had seen or experienced. This is a reasonable strategy for giving the most accurate numeric estimates. But because of this strategy, the numeric scale might have missed the possibility that the context rate shifted participants’ more intuitive, vaguely formed expectations about what the focal gurg would do on the current trial [43,44]. One of the changes introduced in Study 2 is relevant to this issue.

## Study 2

In Study 2, we aimed to replicate the findings from Study 1, with the additional goal of addressing possible concerns about the study design, which we describe below. One of the main issues from Study 1 was that the operationalization of the description-vs.-experience factor may have been insufficient to create the strong experimental difference required for a description-experience gap. Participants in the description condition of that study learned about the hazards not solely based on the description of their explosion rate, but also through the 30 demonstration trials. In Study 2, we strengthened the manipulation by removing the demonstration trials from the learning phase for participants in the description condition. Their learning phase consisted of reading about the summarized risk rates.

A second change was in the time and manner in which we measured likelihood judgments. Recall that we suspected that using the numerical likelihood judgment scale in the latter half of the trials may have led to the lack of contrast effects on likelihood judgments in Study 1. In Study 2, we counterbalanced the order in which likelihood judgments were made—early or late. We also used a verbal likelihood scale instead of a numerical one, since verbal scales may be better at reflecting participants’ intuitive expectations about how hazards would behave [43,44].

Additionally, minor changes were made to the instructions, hazard stimuli, and point system. In Study 2, the hazards took the form of tower buildings instead of gurgs. This change was devised to minimize a potential misinterpretation where participants perceive the gurgs as animate objects that blow up intentionally to hurt them. By using towers instead of gurgs, we could convey to participants that the hazards are inanimate, and the harms afflicted by the hazards are unintentional. There were also changes to the coin amounts and the penalty calculations (see Table 1 Study 2). The instructions were modified to incorporate such changes in the task. However, we did not expect these modifications to be consequential to the overall setup of the DART task nor to the main results.

### Methods

#### Participants and design

The participants (N = 585) were students from introductory psychology courses at the University of Iowa. Participants provided verbal informed consents after reading the consent form approved by the University of Iowa Institutional Review Board and they were recorded via data entry. The written consent was waived by the board. The design was a 2 (Verbal Likelihood Judgment Order: 1st block vs. 3rd block) x 2 (Learning Format: Experience vs. Description) x 2 (Context Risk Rate: 20% vs. 46.7%) x 3 (Coin Amount: 50, 75, or 100) mixed factorial, similar to Study 1. The first three factors were manipulated between participants.

We aimed for a sample size of at least 450. As in Study 1, this target sample size was not based on a formal power analysis but rather a rough estimate of what would provide reasonable power given the prior results and the fact that this study added an order manipulation. In the semester that 450 was reached, we continued data collection (without analyzing data) until the end of the term, because we had a convenient opportunity to boost our sample size given an oversupply of potential research participants in that term. The final sample size of 585 allowed for 80% power to detect a small-to-medium sized contrast effect (*d* = 0.33) within a given learning format condition or to detect a small sized interaction (*f* = 0.11) between learning format and context risk rate [39].

#### Task and procedure

The overall procedure of the task was identical to that of Study 1, except for following modifications.

1. Instead of the gurgs, participants were introduced to two types of abandoned towers that differed in colors. They were instructed that these towers “either violently collapse or harmlessly remain standing.” The violently collapsing of a tower in Study 2 is analogous to explosion of a gurg in Study 1. To avoid confusion, we will continue to use the terms “explosion” and “explosion rate” throughout this paper.
2. The penalties were computed differently from Study 1. Participants caught in the explosion suffered a penalty of 150 and did not get to keep the coins. To balance the resultant change of expected values in the point system, the risky pathway offered a chance to obtain coin amounts that were worth either 50, 75, or 100 rather than 70, 85, or 100 (see Table 1 Study 2).
3. In the learning phase, the distinction between the description and the experience group was purer than in Study 1. That is, for learning about explosion rates, the description group was provided only with the information in a numerical format, whereas the experience group was presented only with the 50 demonstration trials.
4. The numerical likelihood judgments were replaced by verbal likelihood judgments. In the likelihood judgment trials—and prior to making a path decision—participants had to indicate how likely it was that a given tower would collapse (which again, we will refer to as exploding). They used a 7-point response scale that ranged from ‘extremely unlikely’ to ‘extremely likely.’
5. Depending on counterbalancing, participants either made verbal likelihood judgments in the first or third block.
6. Similar to Study 1, there were six unique trial types (i.e., combination of two types of towers and three coin amounts). However, a given trial type was repeated nine times, thus yielding a total of 54 test trials. These repetitions were block randomized across three blocks, such that within one block, all six trial types were presented three times.

### Results

#### Preliminary notes

The analyses reported below focused primarily on the issues of *a priori* interest. Descriptive statistics are provided in the lower panel of Table 2. Given the large sample size and the number of factors involved in the ANOVAs, there were some unanticipated main effects or interactions that reached conventional levels of statistical significance (*p* < .05). However, these effects are only discussed when they would change conclusions about the main issue under investigation and/or had a partial eta squared of at least .01. Full ANOVA tables can be found in the supporting information (S6-S9 Tables in S1 Appendix).

In the following sections, we will only focus on analyses for trials involving focal towers, rather than context towers. As in Study 1, analyses of the context trials revealed that participants were more likely to choose the risky pathway when the tower exploded at a lower rate than at a higher rate. Also, participants took more risks when the coin amount was high rather than low.

As in Study 1, we submitted our proportional data to an arcsine square root transformation [40], and performed identical analyses. The findings were consistent with the results from original data, and thus we report the findings from analysis using the original data. Additionally, a full statistical reporting result of logistic regression can be found in the supporting information (see S10 Table in S1 Appendix).

#### Decisions about focal towers

**Fig 4:**
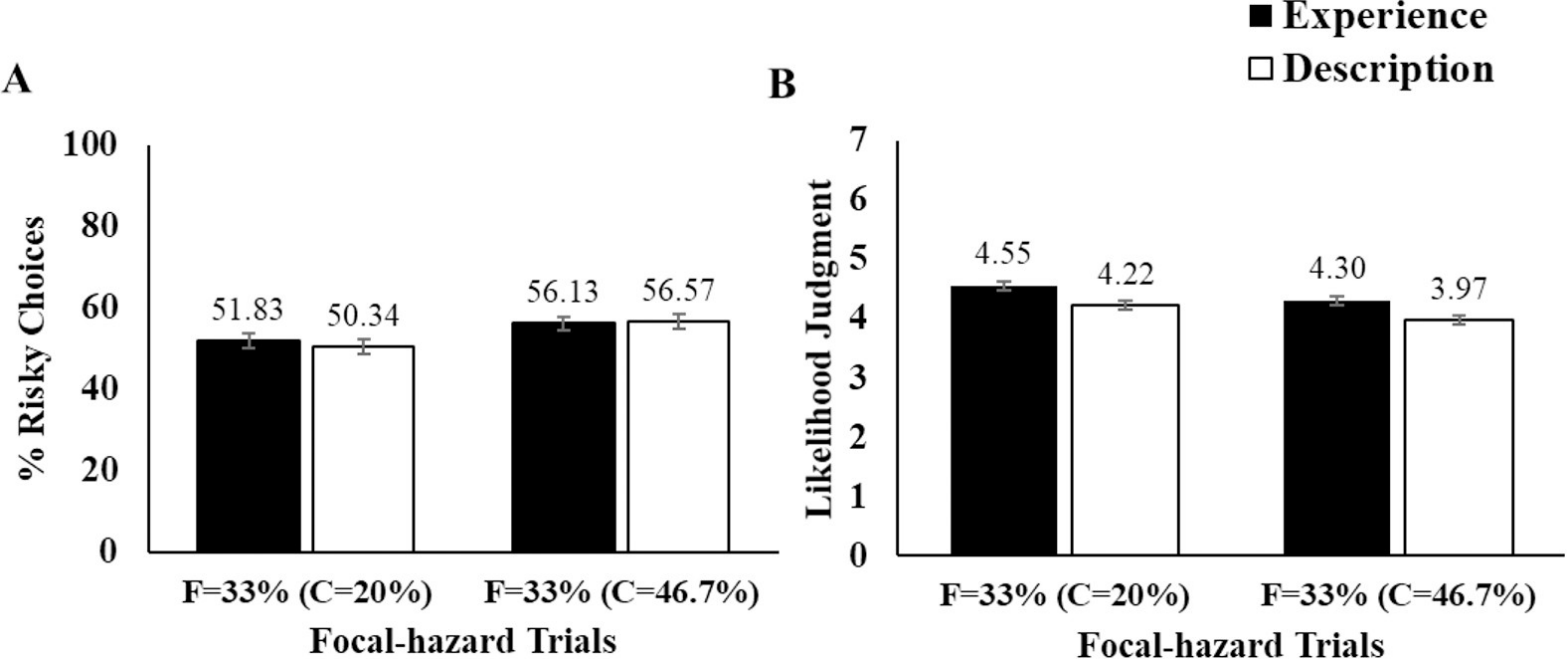
Summary of Study 2 results. C and F indicate risk rates of context and focal hazard, respectively. Note that focal risk rates (i.e., F = 33%) are identical across all context risk rate conditions. (A) Mean proportion of risky choices in focal-hazard trials, (B) Mean likelihood judgment for focal hazards. Black bars indicate the experience condition and white bars indicate the description condition. The numbers above the bars indicate the mean for each condition. Error bar indicates ± 1 S.E.

We submitted risky decision rates from focal trials to a 3 (Coin) x 3 (Block) x 2 (Context Risk Rate) x 2 (Format) x 2 (Likelihood Judgment Block Presentation Order) ANOVA, with the first two factors as repeated measures (Fig 4A). The overall pattern of results replicated the key findings from Study 1. The main effect of the context risk rate was again significant, *F*(1, 576) = 8.90, *p* = .003, *adj* ${\hat{\eta }}_{p}^{2}$ = .02. That is, there was a contrast effect in which participants in the 46.7% context risk rate condition were more likely to choose the risky pathway in focal trials compared to those in the 20% condition. Also as in Study 1, this contrast effect did not significantly differ as a function of format, *F*(1, 576) = .29, *p* = .767. In fact, the contrast effects were remarkably similar in the experience and description conditions. Given the potential importance of this null effect, we computed the Bayes factor for it (*BF*_*01*_ = 15.55). This constitutes strong evidence for the null.

There were no major surprises in the analyses involving coins. Coin amounts again affected decisions, *F*(1.87, 2265.94) = 296.21, *p* < .001, *adj* ${\hat{\eta }}_{p}^{2}$ = .34. Participants chose the risky pathway more when the higher amount of coin was offered. The coin amounts did not interact with either of the key factors; the interaction between coin amounts and learning format was not significant, *F*(1.87, 3019.16) = 2.47, *p* = .089, nor was the interaction between coin amounts and context risk rate, *F*(1.87, 604.63) = .49, *p* = .597.

The effects of block and verbal likelihood judgment order were involved in a number of interactions. Rather than report all of those exhaustively, we will focus on targeted comparisons of most importance. Like Study 1, the impact of the context risk rate was stronger in early trials. The Block x Context Risk Rate was significant, *F*(2, 575) = 8.04, *p* < .001, *adj* ${\hat{\eta }}_{p}^{2}$ =. 027. The three-way interaction term that also included format was not significant, *F*(2, 575) = .181, *p* = .909. When we restricted this type of analysis to just the two “decision-only blocks” (those in which no likelihood judgments were solicited), we saw similar results. The magnitude of the observed contrast effects were stronger when those blocks were at the beginning vs. end of the test phase, *F*(1, 576) = 7.16, *p* = .008, *adj* ${\hat{\eta }}_{p}^{2}$ = .01. More specifically, the contrast effect in the decision-only blocks was robust when those blocks came first, *F*(1, 287) = 14.71, *p* < .001, *adj* ${\hat{\eta }}_{p}^{2}$ = .05, but not when they came last, *F*(1, 289) = .08, *p* = .929. Finally, we also examined the impact of context on decisions in the block that also involved likelihood judgments. For this block, the contrast effect was significant, *F*(1, 576) = 6.55, *p* = .011, *adj* ${\hat{\eta }}_{p}^{2}$ = .01. The magnitude of the contrast effect did not significantly differ as a function of block order, *F*(1, 576) = .06, *p* = .810.

#### Likelihood judgments about focal towers

We calculated the average likelihood judgment per coin level for the trials involving a focal tower. We submitted these values to a 3 (Coin) x 2 (Context Risk Rate) x 2 (Likelihood Judgment Block Presentation Order) x 2 (Format) ANOVA, with the coin factor as a repeated measure (Fig 4B). Unlike in Study 1, we found a significant main effect of context risk rate, *F*(1, 576) = 10.60, *p* = .001, *adj* ${\hat{\eta }}_{p}^{2}$ = .02. Participants in the 20% context risk rate condition judged the focal tower more likely to explode than those in the 46.7% group. The result confirms that the contrast effect may emerge in likelihood judgments as well as in behavioral measures. The magnitude of the contrast effect did not differ as a function of whether likelihood judgments were prompted on trials at the start or at the end of the task, *F*(1, 576) = .20, *p* = .657.

Also, there was a significant main effect of format, where participants in the description group showed lower likelihood judgments than in the experience group, *F*(1, 576) = 18.55, *p* < .001, *adj* ${\hat{\eta }}_{p}^{2}$ = .03. Finally, an unexpected main effect of coin revealed that likelihood judgments were higher for the trials with the higher coin amount compared to the smaller amount *F*(1.89, 2274.37) = 10.86, *p* < .001, *adj* ${\hat{\eta }}_{p}^{2}$ = .02.

### Discussion

The current study replicated the main results from the Study 1. There was a contrast effect on behavioral choices, with participants in the 46.7% context rate group making more risky choices in the focal-tower trials than those in the 20% group. The learning format again did not significantly qualify this contrast effect. It is notable that we again found a similar contrast effect across the two formats even when we used a better manipulation of the description-vs.-experience factor. Unlike Study 1, the learning phase for participants in the description condition started with participants viewing summarized numerical information about the hazards’ explosion rate; there were no iterative learning trials in the learning phase of the description condition.

Analyses also revealed that, whereas the contrast effect was robust immediately at the start of the test phase (and was significant in analyses that collapsed across all trials), it tended to weaken by the final block. Exactly why it weakened is uncertain. One possibility is that repeatedly experiencing the costs or benefits of making decisions about specific hazards helped people ignore irrelevant across-hazard comparisons. The implications of this order effect are addressed more in the General Discussion.

Another noteworthy finding is that, unlike Study 1, we found a contrast effect in participants’ likelihood judgments for focal towers. Participants in the 20% context rate group judged the focal towers to be more likely to explode compared to those in the 46.7% group, which matches the contrast effect observed in their behavioral choices. The difference between the findings from Studies 1 and 2 might be partially due to the placement of the block that included likelihood judgments in the trials. In Study 1, this block was always at the very end of the study, whereas in Study 2, the block was counterbalanced to appear either at the beginning or at the end of the study. However, we suspect that an important reason for the difference in findings concerns how we solicited likelihood judgments. Participants in Study 2 were prompted to assess the perceived riskiness of each hazard on a scale marked with verbal expressions of chance (i.e., extremely unlikely—extremely likely) rather than the numerical anchors used in Study 1 (i.e., 0% - 100%). The numeric scale may have encouraged a response strategy that precludes sensitivity to the contextual effects [11,43–45]. Participants using the numeric scale in Study 1 may have deliberatively dwelled on proportion information they had seen or experienced when estimating likelihood. Knowledge of this proportion information might remain intact and unbiased by context, even as participants’ intuitions about what is likely to happen on a trial shift as a function of context. Various studies have shown that verbal scales are sometimes better at detecting contextual factors that influence intuitive impressions of uncertainty, and these intuitive impressions are often key drivers of decisions and behaviors [11,43–46]. If the results of Study 2 were not available, the lack of contrast effects on likelihood judgments in Study 1 might be interpreted as suggesting that the contrast effects in behavior were a mere consequence of a behavioral strategy that did not involve any distortions of the perceived riskiness of hazards. The significant contrast effect on likelihood judgments in Study 2 casts doubt on that interpretation and suggests that there were important distortions in the perceived riskiness of the focal hazard.

Lastly, we will note two additional findings regarding likelihood judgments. First, as in Study 1, participants in the experience group gave higher likelihood judgments than did those in the description group. More about this interesting but tangential finding will be said in the General Discussion. Second, there was an unexpected finding involving coin amounts that is also intriguing, albeit not particularly germane to the rest of the paper. Participants judged the towers to be more risky in the trials that offered larger (vs. smaller) coin amounts. Exactly why this happened is unknown. The effect might be related to the risk-reward heuristic [47,48], where people infer the level of risk from the magnitude of the payoffs offered, which tend to correlate positively in many environments. The effect was not present in Study 1, so it might be unique to intuitive risk impressions that are tapped by verbal likelihood measures.

## General discussion

The main goal of the project was to determine how the format in which people learn about the riskiness of hazards affects the magnitude and the direction of context effects. Across two experiments, we investigated context effects that biased the perceived riskiness of hazards and the responses to them. Using the DART, we measured participants’ responses to hazards in a dynamic setup that is analogous to everyday decision contexts where people learn and make decisions about potential risks. In both studies, the learning format and the risk rates of the context hazards were manipulated. Study 1 revealed reliable contrast effects in behavioral responses; participants made more risky choices for focal hazards when the context risk rate was high compared to when it was low. However, this effect did not vary as a function of the learning format. For Study 2, we refined the learning-format manipulation to instantiate a clearer distinction between the description and experience condition. Nevertheless, the results replicated the main findings from Study 1; the behavioral responses again revealed a significant contrast effect that was not moderated by the learning format.

The results for likelihood judgments were slightly different across Studies 1 and 2, but this was likely due to methodological changes that we made in Study 2 to address potential weaknesses in Study 1. In Study 2, a contrast effect was observed in people’s likelihood judgments consistent with the contrast effect found in their behavior, whereas we did not find such consistency in Study 1. Among the changes we introduced in Study 2, we suspect that using a verbal scale for soliciting likelihood judgments (instead of a numerical one) played a central role. The verbal scale, but not the numeric one, was effective at capturing how participant’s intuitive impressions about the focal hazards’ riskiness were distorted by the risk level of the contextual hazard [45,46].

As discussed in the Introduction, the contrast effect observed in DFD can be explained in a variety of ways, many of which are interrelated. First, it is possible that in DFD, contrast effects could be driven by the categorization or gist-like evaluation of the focal hazard relative to the distinct context hazard [22–27]. Second, a selective accessibility account would suggest that the explicit description of riskiness would facilitate a selective search for dissimilarity between the focal and context hazards to confirm the discrepancy hypothesis (26). Third, decision-by-sampling highlights that riskiness of the hazards are compared in a pairwise fashion, which would involve assessments based on the differences in hazards’ riskiness, resulting in a contrast effect [30,49]. Lastly, according to range-frequency theory, focal hazards would be assessed in light of how the range and frequency of the context attributes are distributed [29]. Different context risk rates would generate different context distributions, altering the position on which the focal hazard would be mapped.

Although each of these accounts have the potential to explicate the contrast effects observed in the DFD format within our DART paradigm, not all of them would predict similar contrast effects in the DFE format. If we had observed either a null effect or an assimilation effect in our DFE condition rather than a contrast effect, such results would have favored some perspectives over others. We discussed three such accounts in the Introduction. First, the selective accessibility account [28] is one that could have been applied to explain a description-experience gap. According to the account, the dissimilarities between the context and focal hazards would not be distinguished immediately in DFE as in DFD, because the risk levels for the hazards have to be learned over repeated experiences. Because the overall difference between the hazards would not be detected immediately, selective search for the instances that confirm the distinction between the two hazards would be also unlikely to occur, ultimately causing a smaller (or absent or reversed) context effect in DFE than in DFD. The categorization account is a second approach that would have been favored if a description-experience gap had been detected [24–27]. According to this account, people would have initial difficulty in categorizing the riskiness of the hazards more in DFE than in DFD. This is because such categorization would require at least some repeated exposure to the hazards in order to generate impressions about them. Third, we also mused about the possibility that an assimilation effect might occur in DFE (but not DFD) due to source confusion [34], although we deemed this to be less likely.

Given that we found equivalently sized contrast effects in DFD and DFE, the findings favor accounts whose mechanisms would presumably operate similarly in DFD and DFE. Both decision-by-sampling [30,49] and range-frequency theory [29] fit this description. Again, decision-by-sampling posits pairwise comparisons between the value of a focal attribute and a value from a context distribution. Specifically, the context distribution includes both information present at the time of assessment, but also knowledge from past encounters and experiences. Therefore, even when the explicit descriptions of the attributes—such as explosion rates of hazards—are unavailable, the accumulated experience of attribute values can lead to a contrast effect because one of these values could be included in a pairwise comparison with of the focal-attribute value. Likewise, range-frequency theory could account for similar contrast effects in the two learning formats. Both range and frequency distributions can be generated from repeated experiences and descriptive information. In turn, the focal hazard would be assessed based on the range and frequency distribution that is in essence the same, regardless of the learning format. Additionally, the contrast effect in DFE is consistent with what has been suggested by neurophysiological research regarding the contextual modulation of value [36–38]. In summary, decision-by-sampling, range-frequency theory and evidence supporting the contextual value adaptation all successfully account for the context effects found across DFD and DFE, whereas some theoretical approaches such as the categorization or selective accessibility accounts do not.

### Comparing the description-experience gaps

Our findings produced no evidence of a description-experience gap, whereas other studies have found reliable gaps (for review see [15]). We do not consider this to be an inconsistency in the literature because the type of gap tested in our studies was conceptually quite different from the most prominently discussed description-experience gap [2,3]. In the widely-researched gap, the focus is on the under- vs. over-weighting of the rare events, whereas our focus was on the biasing influence of context.

More closely related to our findings are those from studies that investigated decoy effects in the description-experience gap. Ert and Lejarraga [16] found that the decoy effect was observed only in a DFD condition, and not in DFE. The authors suggested that the decoy effect in DFD arises when people are consciously aware of the distinction between a target and decoy, that creates an asymmetric dominance. In DFE, however, it may be hard for people to notice a subtle distinction between the target and the decoy option, which obviates the possibility for a decoy effect [16]. Indeed, when participants were able to identify the dominance relationship between the options, those making DFE also exhibited a decoy effect similar to those in the DFD condition [16,18].

These finding raises the possibility that a description-experience gap in our paradigm might be more likely to be observed if the context rates were more similar to the focal rates. Imagine, for example, that the focal and context rates differed only by a few percentage points. In DFD, this difference would still be easily noticed and could produce contrast effects. However, in DFE, participants might never notice this difference and therefore the DFE condition might never produce reliable contrast effects.

### Additional findings

#### Change in contrast effect across blocks

Thus far, we have said little about another feature of the contrast effects detected in the DART studies. Namely, the contrast effects were robust in early blocks and faded over the latter blocks, and this pattern was not moderated by learning format. The fact that the contrast effects in the DFD and DFE condition were highly similar even immediately after the learning phase is notable. It removes a concern that the lack of differences (regrading contrast effects) between the DFD and DFE conditions was because both conditions have similar experiences within the test blocks. The general attenuation of the contrast effects across blocks could have various causes, but we suspect that the repeated valenced feedback per trial played a role. By “valenced feedback” we are referring to the fact that witnessing the outcome of each trial gave people positive or negative feedback—i.e., they either did or did not have a proper expectation about (and made a good decision in light of) whether the hazard would or would not cause harm. This self-relevant, valenced feedback throughout the test trials might have helped correct biased expectations that they held in earlier trials.

#### Main effect of learning format in likelihood judgments

An interesting, albeit tangential finding regarding likelihood judgments in both studies was a main effect of learning format. Participants gave lower likelihood judgments when they had learned about a hazard via description than via experience. Such finding is at odds with the fact that there was no description-experience gap in risky choices. A potential reason for this discrepancy in description-experience gap across the measures may be that likelihood judgments were differentially impacted by numeric anchors [50] depending on the learning format, whereas risky choices were not impacted by anchors.

Unlike participants in DFD conditions, those in DFE conditions were not explicitly given information that could work as a convenient initial anchor—such as explicit numerical descriptions of the hazards’ riskiness—which can be mapped onto the numerical and verbal likelihood scale relatively easily. Given the lack of such numerical anchors, combined with a high level of uncertainty in the actual risk rate, participants in the DFE condition may have relied on arbitrary mid-range probabilities as a possible starting point for their responses. An insufficient adjustment from this mid-range starting point may be why we observed overestimation in likelihood judgments from those in the DFE condition compared to the DFD condition. On the other hand, risky choices would be less affected by an anchoring effect compared to likelihood judgments, given that risky choices require dichotomous, rather than continuous responses.

### Limitations

The current studies are not without limitations. Perhaps the most notable limitation is that we used the same probability levels (i.e., the rates at which the hazards caused harm) in both studies. The focal probability was always 33% and the context rates were either 20% or 46.7%–exposing participants to hazards that varied only in two levels of riskiness. Despite being restrictive, this was an intentional choice aimed at creating differences between contexts and focal rates that could be noticed and learned. We also note that these focal and context rates kept participants away from floor and ceiling effects in their patterns of responses. Furthermore, variation in the coin amount—combined with the two risk rates—created a diverse set of expected values associated with risky and safe choices, keeping the participants’ decision making task from becoming monotonous. With all this said, we acknowledge that future research could greatly benefit from investigating how the response would change when a wider range of options are included.

A second limitation is that the current task did not include a baseline condition in which responses to the focal hazard were measured in isolation (i.e. in the absence of any context hazard). Having a baseline condition involving only the focal hazard trials would have allowed direct tests for context effects within each cell, which allows for symmetry claims, which we did not make here (see [51]). It is technically possible that risky choice rates in a baseline condition would be higher than in both of the context conditions, but this seems highly implausible. Even if this possibility was true, we would still be left with the fact that risky choice rates were higher when the context hazard rates were higher (vs. lower) than the focal hazard rate—which is a direction consistent with contrast and inconsistent with assimilation.

## Conclusion

The information people need to make appropriate risky decisions is rarely straightforward. Across many domains (e.g., medical, financial) people are required to compare a wide range of risky options learned from diverse sources. Because real-life risky decisions often involve threats or hazards about which people might make comparisons, it is crucial that we understand how people incorporate and compare risk information acquired about different threats and involving different formats of information. Our project used the DART paradigm as a means of instantiating some of the complexities of decision making environments while keeping a strong conceptual connection with well-established classical gambling paradigms. With DART, we were able to examine how contextual information influences perceptions of and responses to specific risks, and we directly compared these influences under two important learning formats. We found contexts effects that fell in the direction of contrast rather than assimilation. Importantly, in both experiments, these contrast effects did not differ whether the information about the hazards was acquired from experience or from description. These findings favor theories of context effects that posit mechanisms that are broad-based and would operate similarly under learning from description vs. experience.

## Supporting information

S1 Appendix (DOCX)
