# Supplementary material for: Context dependency in risky decision making: Is there a description-experience gap?
Source: PLoS One. 2021 Feb 11;16(2):e0245969. doi: 10.1371/journal.pone.0245969 (PMC7877666; doi:10.1371/journal.pone.0245969)
Supplement: S1 Appendix — (DOCX) [file pone.0245969.s001.docx]

**Supporting Information**

**Mixed-ANOVA result for Study 1**

*Note.* Abbreviation used in the following tables are SS= Sum of Square; df = Degree of freedom; MS = Mean Sum of Square.

**S1 Table. Mixed ANOVA on % risky choices for context gurgs from Study 1**

| Predictor | *SS* | *df* | *MS* | *F* | *p* | *partial η^2^* |
| --- | --- | --- | --- | --- | --- | --- |
| Context Risk Rate | 80330.54 | 1 | 80330.54 | 42.59 | <.001 | 0.12 |
| Learning Format | 345.70 | 1 | 345.70 | 0.18 | 0.669 | <.001 |
| Context Risk Rate * Learning Format | 1311.29 | 1 | 1311.29 | 0.70 | 0.405 | <.001 |
| Block | 11.05 | 1 | 11.05 | 0.02 | 0.884 | <.001 |
| Block * Context Risk Rate | 158.77 | 1 | 158.77 | 0.31 | 0.579 | <.001 |
| Block * Learning Format | 363.27 | 1 | 363.27 | 0.71 | 0.401 | <.001 |
| Block * Context Risk Rate * Learning Format | 602.56 | 1 | 602.56 | 1.17 | 0.280 | <.001 |
| Coin | 90329.52 | 2 | 45164.76 | 83.38 | <.001 | 0.22 |
| Coin * Context Risk Rate | 2404.41 | 2 | 1202.20 | 2.22 | 0.110 | 0.01 |
| Coin * Learning Format | 2906.86 | 2 | 1453.43 | 2.68 | 0.069 | 0.01 |
| Coin * Context Risk Rate * Learning Format | 1334.77 | 2 | 667.39 | 1.23 | 0.292 | <.001 |
| Block * Coin | 472.81 | 2 | 236.41 | 0.60 | 0.550 | <.001 |
| Block * Coin * Context Risk Rate | 1539.09 | 2 | 769.55 | 1.95 | 0.144 | 0.01 |
| Block * Coin * Learning Format | 128.37 | 2 | 64.19 | 0.16 | 0.850 | <.001 |
| Block * Coin * Context Risk Rate * Learning Format | 2420.51 | 2 | 1210.25 | 3.06 | 0.048 | 0.01 |

**S2 Table. Mixed ANOVA on % risky choices for focal gurgs from Study 1**

| Predictor | *SS* | *df* | *MS* | *F* | *p* | *partial η2* |
| --- | --- | --- | --- | --- | --- | --- |
| Context Risk Rate | 29956.69 | 1 | 29956.69 | 19.25 | <.001 | 0.06 |
| Learning Format | 433.63 | 1 | 433.63 | 0.28 | 0.598 | <.001 |
| Context Risk Rate * Learning Format | 4848.61 | 1 | 4848.61 | 3.12 | 0.079 | 0.01 |
| Block | 43.30 | 1 | 43.30 | 0.09 | 0.769 | <.001 |
| Block * Context Risk Rate | 2719.07 | 1 | 2719.07 | 5.45 | 0.020 | 0.02 |
| Block * Learning Format | 10.49 | 1 | 10.49 | 0.02 | 0.885 | <.001 |
| Block * Context Risk Rate * Learning Format | 271.89 | 1 | 271.89 | 0.55 | 0.461 | <.001 |
| Coin | 122669.63 | 2 | 61334.82 | 94.02 | <.001 | 0.24 |
| Coin * Context Risk Rate | 1060.16 | 2 | 530.08 | 0.81 | 0.444 | <.001 |
| Coin * Learning Format | 29.62 | 2 | 14.81 | 0.02 | 0.978 | <.001 |
| Coin * Context Risk Rate * Learning Format | 163.70 | 2 | 81.85 | 0.13 | 0.882 | <.001 |
| Block * Coin | 1239.69 | 2 | 619.84 | 1.30 | 0.272 | <.001 |
| Block * Coin * Context Risk Rate | 539.78 | 2 | 269.89 | 0.57 | 0.567 | <.001 |
| Block * Coin * Learning Format | 3437.26 | 2 | 1718.63 | 3.62 | 0.027 | 0.01 |
| Block * Coin * Context Risk Rate * Learning Format | 580.07 | 2 | 290.03 | 0.61 | 0.544 | <.001 |

**S3 Table. Mixed ANOVA on likelihood judgment for context gurgs from Study 1**

| Predictor | *SS* | *df* | *MS* | *F* | *p* | *partial η2* |
| --- | --- | --- | --- | --- | --- | --- |
| Context Risk Rate | 32854.86 | 1 | 32854.86 | 64.88 | <.001 | 0.18 |
| Learning Format | 20094.65 | 1 | 20094.65 | 39.68 | <.001 | 0.12 |
| Context Risk Rate * Learning Format | 1403.15 | 1 | 1403.15 | 2.77 | 0.097 | 0.01 |
| Coin | 275.76 | 2 | 137.88 | 0.84 | 0.434 | <.001 |
| Coin * Context Risk Rate | 203.88 | 2 | 101.94 | 0.62 | 0.539 | <.001 |
| Coin * Learning Format | 291.72 | 2 | 145.86 | 0.89 | 0.413 | <.001 |
| Coin * Context Risk Rate * Learning Format | 430.59 | 2 | 215.29 | 1.31 | 0.271 | <.001 |

**S4 Table. Mixed ANOVA on likelihood judgment for focal gurgs from Study 1**

| Predictor | *SS* | *df* | *MS* | *F* | *p* | *partial η2* |
| --- | --- | --- | --- | --- | --- | --- |
| Context Risk Rate | 855.61 | 1 | 855.61 | 1.92 | 0.167 | 0.01 |
| Learning Format | 14423.84 | 1 | 14423.84 | 32.31 | <.001 | 0.10 |
| Context Risk Rate * Learning Format | 117.10 | 1 | 117.10 | 0.26 | 0.609 | <.001 |
| Coin | 389.35 | 2 | 194.68 | 0.99 | 0.371 | <.001 |
| Coin * Context Risk Rate | 61.71 | 2 | 30.85 | 0.16 | 0.854 | <.001 |
| Coin * Learning Format | 191.33 | 2 | 95.66 | 0.49 | 0.614 | <.001 |
| Coin * Context Risk Rate * Learning Format | 204.40 | 2 | 102.20 | 0.52 | 0.594 | <.001 |

**Logistic Regression for Study 1**

Logistic mixed-effects regression analyses were conducted for context and focal tower trials separately, with risky choice as a dependent variable (safe choice coded as 0 and risky choice coded as 1), context risk rate, learning format and coin as fixed effects, and participant as a random effect (S5 Table). The analyses were performed via the R software using the glmer function in lme4 package [1].

Consistent with the results from mixed-ANOVA analyses, context risk rate (*B* = -0.33, *z* = -7.03, *p* < .001) and coin (*B* = -0.03, *z* = 14.27, *p* < .001) were significant predictors predicting the changes in likelihood of risky choices in context gurg trials. Similarly, in focal gurg trials, context risk rate (*B* = 0.19, *z* = 4.41, *p* < .001) and coin (*B* = 0.03, *z* = 16.41, *p* < .001) were significant predictors predicting the changes in likelihood of risky choices. In both context and focal gurg trials, learning format did not significantly predict changes in likelihood of risky choices.

**S5 Table. Logistic mixed regression on % risky choices for context and focal gurgs from Study 1**

|  | Predictor | Variance | *B* | *Std. error* | *z* | *p* |
| --- | --- | --- | --- | --- | --- | --- |
| Context Gurg Trials | Random effects |  |  |  |  |  |
|  | RP (Intercept) | 0.30 |  | 0.54 |  |  |
|  | Fixed effects |  |  |  |  |  |
|  | (Intercept) |  | 0.53 | 0.05 | 11.14 | < .001*** |
|  | Learning Format |  | < 0.001 | 0.05 | 0.00 | 1.00 |
|  | Context Risk Rate |  | -0.33 | 0.05 | -7.03 | < .001*** |
|  | Coin |  | 0.03 | 0.00 | 14.27 | < .001*** |
|  | Learning Format*Context Risk Rate |  | 0.04 | 0.05 | 0.87 | 0.385 |
|  | Learning Format*Coin |  | < 0.001 | 0.00 | -2.04 | 0.041* |
|  | Context Risk Rate*Coin |  | < 0.001 | 0.00 | -0.52 | 0.602 |
|  | Learning Format*Context Risk Rate*Coin |  | < 0.001 | 0.00 | -0.10 | 0.919 |
| Focal Gurg Trials | Predictor | Variance | *B* | *Std. error* | *z* | *p* |
|  | Random effects |  |  |  |  |  |
|  | RP (Intercept) | 0.22 |  | 0.46 |  |  |
|  | Fixed effects |  |  |  |  |  |
|  | Intercept |  | 0.56 | 0.04 | 13.27 | < .001*** |
|  | Learning Format |  | -0.03 | 0.04 | -0.78 | 0.435 |
|  | Context Risk Rate |  | 0.19 | 0.04 | 4.41 | < .001*** |
|  | Coin |  | 0.03 | 0.00 | 16.41 | < .001*** |
|  | Learning Format*Context Risk Rate |  | -0.08 | 0.04 | -1.81 | 0.071 |
|  | Learning Format*Coin |  | < 0.001 | 0.00 | -0.29 | 0.773 |
|  | Context Risk Rate*Coin |  | < 0.001 | 0.00 | -0.90 | 0.366 |
|  | Learning Format*Context Risk Rate*Coin |  | < 0.001 | 0.00 | 0.14 | 0.889 |

*Note*. Asterisks indicate significance at p < 0.05 (*), p < 0.01 (**) and p < 0.001 (***)

**Mixed-ANOVA result for Study 2**

*Note.* Abbreviation used in the following tables are SS= Sum of Square; df = Degree of freedom; MS = Mean Sum of Square.

**S6 Table. Mixed ANOVA on % risky choices for context towers from Study 2**

| Predictor | *SS* | *df* | *MS* | *F* | *p* | *partial η2* |
| --- | --- | --- | --- | --- | --- | --- |
| Context Risk Rate | 484746.83 | 1 | 484746.83 | 115.77 | <.001 | 0.17 |
| Block Order CB | 4146.58 | 1 | 4146.58 | 0.99 | 0.320 | <0.01 |
| Learning Format | 3489.98 | 1 | 3489.98 | 0.83 | 0.362 | <0.01 |
| Context Risk Rate * Block Order CB | 178.74 | 1 | 178.74 | 0.04 | 0.836 | <0.01 |
| Context Risk Rate * Learning Format | 3648.69 | 1 | 3648.69 | 0.87 | 0.351 | <0.01 |
| Block Order CB * Learning Format | 4094.68 | 1 | 4094.68 | 0.98 | 0.323 | <0.01 |
| Context Risk Rate * Block Order CB * Learning Format | 85.32 | 1 | 85.32 | 0.02 | 0.887 | <0.01 |
| Block | 10860.97 | 2 | 5430.49 | 6.79 | 0.001 | 0.01 |
| Block * Context Risk Rate | 8519.16 | 2 | 4259.58 | 5.33 | 0.005 | 0.01 |
| Block * Block Order CB | 10961.02 | 2 | 5480.51 | 6.85 | 0.001 | 0.01 |
| Block * Learning Format | 1242.08 | 2 | 621.04 | 0.78 | 0.460 | <0.01 |
| Block * Context Risk Rate * Block Order CB | 5490.80 | 2 | 2745.40 | 3.43 | 0.033 | 0.01 |
| Block * Context Risk Rate * Learning Format | 291.23 | 2 | 145.61 | 0.18 | 0.834 | <0.01 |
| Block * Block Order CB * Learning Format | 4605.51 | 2 | 2302.76 | 2.88 | 0.057 | 0.01 |
| Block * Context Risk Rate * Block Order CB * Learning Format | 45.16 | 2 | 22.58 | 0.03 | 0.972 | <0.01 |
| Error(Block) | 921313.46 | 1152 | 799.75 |  |  |  |
| Coin | 608353.49 | 1.86 | 327870.16 | 265.60 | <.001 | 0.32 |
| Coin * Context Risk Rate | 9928.52 | 1.86 | 5350.94 | 4.34 | 0.015 | 0.01 |
| Coin * Block Order CB | 9985.06 | 1.86 | 5381.42 | 4.36 | 0.015 | 0.01 |
| Coin * Learning Format | 1669.45 | 1.86 | 899.75 | 0.73 | 0.473 | <0.01 |
| Coin * Context Risk Rate * Block Order CB | 1390.09 | 1.86 | 749.19 | 0.61 | 0.533 | <0.01 |
| Coin * Context Risk Rate * Learning Format | 867.68 | 1.86 | 467.63 | 0.38 | 0.669 | <0.01 |
| Coin * Block Order CB * Learning Format | 2125.11 | 1.86 | 1145.32 | 0.93 | 0.390 | <0.01 |
| Coin * Context Risk Rate * Block Order CB * Learning Format | 705.26 | 1.86 | 380.10 | 0.31 | 0.719 | <0.01 |
| Error(Coin) | 1319335.98 | 1068.75 | 1234.47 |  |  |  |
| Block * Coin | 14734.45 | 4 | 3683.61 | 5.92 | <.001 | 0.01 |
| Block * Coin * Context Risk Rate | 544.10 | 4 | 136.03 | 0.22 | 0.928 | <0.01 |
| Block * Coin * Block Order CB | 22222.01 | 4 | 5555.50 | 8.93 | 0.000 | 0.02 |
| Block * Coin * Learning Format | 2353.77 | 4 | 588.44 | 0.95 | 0.436 | <0.01 |
| Block * Coin * Context Risk Rate * Block Order CB | 2127.35 | 4 | 531.84 | 0.86 | 0.490 | <0.01 |
| Block * Coin * Context Risk Rate * Learning Format | 3839.96 | 4 | 959.99 | 1.54 | 0.187 | <0.01 |
| Block * Coin * Block Order CB * Learning Format | 331.90 | 4 | 82.97 | 0.13 | 0.970 | <0.01 |
| Block* Coin* Context Risk Rate*Block Order CB*Learning Format | 2206.81 | 4 | 551.70 | 0.89 | 0.471 | <0.01 |
| Error(Block*Coin) | 1433036.80 | 2304 | 621.98 |  |  |  |

**S7 Table. Mixed ANOVA on % risky choices for focal towers from Study 2**

| Predictor | *SS* | *df* | *MS* | *F* | *p* | *partial η2* |
| --- | --- | --- | --- | --- | --- | --- |
| Context Risk Rate | 36467.93 | 1 | 36467.93 | 8.90 | 0.003 | 0.02 |
| Block Order CB | 1139.24 | 1 | 1139.24 | 0.28 | 0.598 | <0.01 |
| Learning Format | 360.61 | 1 | 360.61 | 0.09 | 0.767 | <0.01 |
| Context Risk Rate * Block Order CB | 16028.40 | 1 | 16028.40 | 3.91 | 0.048 | 0.01 |
| Context Risk Rate * Learning Format | 1230.90 | 1 | 1230.90 | 0.30 | 0.584 | <0.01 |
| Block Order CB * Learning Format | 6.42 | 1 | 6.42 | 0.00 | 0.968 | <0.01 |
| Context Risk Rate * Block Order CB * Learning Format | 5492.74 | 1 | 5492.74 | 1.34 | 0.247 | <0.01 |
| Block | 10542.37 | 2 | 5271.19 | 6.41 | 0.002 | 0.01 |
| Block * Context Risk Rate | 12492.19 | 2 | 6246.09 | 7.60 | 0.001 | 0.01 |
| Block * Block Order CB | 21030.41 | 2 | 10515.20 | 12.79 | <0.001 | 0.02 |
| Block * Learning Format | 6625.97 | 2 | 3312.98 | 4.03 | 0.018 | 0.01 |
| Block * Context Risk Rate * Block Order CB | 5908.99 | 2 | 2954.50 | 3.59 | 0.028 | 0.01 |
| Block * Context Risk Rate * Learning Format | 1591.03 | 2 | 795.52 | 0.97 | 0.38 | <0.01 |
| Block * Block Order CB * Learning Format | 626.24 | 2 | 313.12 | 0.38 | 0.683 | <0.01 |
| Block * Context Risk Rate * Block Order CB * Learning Format | 1153.04 | 2 | 576.52 | 0.70 | 0.496 | <0.01 |
| Error(Block) | 947160.99 | 1152.00 | 822.19 |  |  |  |
| Coin | 676632.61 | 1.87 | 362540.93 | 296.21 | <0.001 | 0.34 |
| Coin * Context Risk Rate | 1128.45 | 1.87 | 604.63 | 0.49 | 0.597 | <0.01 |
| Coin * Block Order CB | 4313.54 | 1.87 | 2311.20 | 1.89 | 0.155 | <0.01 |
| Coin * Learning Format | 5634.87 | 1.87 | 3019.18 | 2.47 | 0.089 | <0.01 |
| Coin * Context Risk Rate * Block Order CB | 4906.78 | 1.87 | 2629.06 | 2.15 | 0.121 | <0.01 |
| Coin * Context Risk Rate * Learning Format | 0.40 | 1.87 | 0.21 | 0.00 | 1 | <0.01 |
| Coin * Block Order CB * Learning Format | 3313.56 | 1.87 | 1775.41 | 1.45 | 0.235 | <0.01 |
| Coin * Context Risk Rate * Block Order CB * Learning Format | 4124.22 | 1.87 | 2209.77 | 1.81 | 0.168 | <0.01 |
| Error(Coin) | 1315762.44 | 1075.03 | 1223.94 |  |  |  |
| Block * Coin | 17342.23 | 3.93 | 4408.38 | 6.69 | <0.001 | 0.01 |
| Block * Coin * Context Risk Rate | 2935.04 | 3.93 | 746.09 | 1.13 | 0.34 | <0.01 |
| Block * Coin * Block Order CB | 24584.22 | 3.93 | 6249.30 | 9.48 | <0.001 | 0.02 |
| Block * Coin * Learning Format | 844.08 | 3.93 | 214.56 | 0.33 | 0.858 | <0.01 |
| Block * Coin * Context Risk Rate * Block Order CB | 959.37 | 3.93 | 243.87 | 0.37 | 0.827 | <0.01 |
| Block * Coin * Context Risk Rate * Learning Format | 4872.69 | 3.93 | 1238.64 | 1.88 | 0.113 | <0.01 |
| Block * Coin * Block Order CB * Learning Format | 2364.62 | 3.93 | 601.09 | 0.91 | 0.455 | <0.01 |
| Block*Coin*Context Risk Rate*Block Order CB*Learning Format | 1160.87 | 3.93 | 295.09 | 0.45 | 0.771 | <0.01 |
| Error(Block*Coin) | 1494317.76 | 2265.94 | 659.47 |  |  |  |

**S8 Table. Mixed ANOVA on likelihood judgment for context towers from Study 2**

| Predictor | *SS* | *df* | *MS* | *F* | *p* | *partial η2* |
| --- | --- | --- | --- | --- | --- | --- |
| Context Risk Rate | 840.30 | 1 | 840.30 | 106.95 | <0.001 | 0.16 |
| Block Order CB | 1.29 | 1 | 1.29 | 0.17 | 0.685 | <0.01 |
| Learning Format | 41.43 | 1 | 41.43 | 5.27 | 0.022 | 0.01 |
| Context Risk Rate * Block Order CB | 0.19 | 1 | 0.19 | 0.03 | 0.876 | <0.01 |
| Context Risk Rate * Learning Format | 17.35 | 1 | 17.35 | 2.21 | 0.138 | <0.01 |
| Block Order CB * Learning Format | 32.47 | 1 | 32.47 | 4.13 | 0.043 | 0.01 |
| Context Risk Rate * Block Order CB * Learning Format | 7.38 | 1 | 7.38 | 0.94 | 0.333 | <0.01 |
| Coin | 15.46 | 1.87 | 8.26 | 6.53 | 0.002 | 0.01 |
| Coin * Context Risk Rate | 0.51 | 1.87 | 0.27 | 0.21 | 0.793 | <0.01 |
| Coin * Block Order CB | 0.65 | 1.87 | 0.35 | 0.27 | 0.746 | <0.01 |
| Coin * Learning Format | 1.22 | 1.87 | 0.65 | 0.52 | 0.586 | <0.01 |
| Coin * Context Risk Rate * Block Order CB | 1.34 | 1.87 | 0.71 | 0.56 | 0.558 | <0.01 |
| Coin * Context Risk Rate * Learning Format | 0.63 | 1.87 | 0.34 | 0.27 | 0.753 | <0.01 |
| Coin * Block Order CB * Learning Format | 4.24 | 1.87 | 2.27 | 1.79 | 0.170 | <0.01 |
| Coin * Context Risk Rate * Block Order CB * Learning Format | 1.35 | 1.87 | 0.72 | 0.57 | 0.554 | <0.01 |
| Error(Coin) | 1363.25 | 1077.96 | 1.27 |  |  |  |

**S9 Table. Mixed ANOVA on likelihood judgment for focal towers from Study 2**

| Predictor | *SS* | *df* | *MS* | *F* | *p* | *partial η^2^* |
| --- | --- | --- | --- | --- | --- | --- |
| Context Risk Rate | 82.64 | 1 | 82.64 | 10.60 | 0.001 | 0.02 |
| Block Order CB | 3.20 | 1 | 3.20 | 0.41 | 0.522 | <0.01 |
| Learning Format | 144.56 | 1 | 144.56 | 18.55 | <.001 | 0.03 |
| Context Risk Rate * Block Order CB | 1.54 | 1 | 1.54 | 0.20 | 0.657 | <0.01 |
| Context Risk Rate * Learning Format | 0.01 | 1 | 0.01 | 0.00 | 0.971 | <0.01 |
| Block Order CB * Learning Format | 21.25 | 1 | 21.25 | 2.73 | 0.099 | 0.01 |
| Context Risk Rate * Block Order CB * Learning Format | 4.50 | 1 | 4.50 | 0.58 | 0.448 | <0.01 |
| Coin | 26.69 | 1.89 | 14.15 | 10.86 | <.001 | 0.02 |
| Coin * Context Risk Rate | 5.46 | 1.89 | 2.89 | 2.22 | 0.112 | <0.01 |
| Coin * Block Order CB | 1.30 | 1.89 | 0.69 | 0.53 | 0.579 | <0.01 |
| Coin * Learning Format | 0.52 | 1.89 | 0.28 | 0.21 | 0.796 | <0.01 |
| Coin * Context Risk Rate * Block Order CB | 0.58 | 1.89 | 0.31 | 0.24 | 0.777 | <0.01 |
| Coin * Context Risk Rate * Learning Format | 5.41 | 1.89 | 2.87 | 2.20 | 0.114 | <0.01 |
| Coin * Block Order CB * Learning Format | 2.14 | 1.89 | 1.13 | 0.87 | 0.414 | <0.01 |
| Coin * Context Risk Rate * Block Order CB * Learning Format | 3.78 | 1.89 | 2.00 | 1.54 | 0.217 | <0.01 |
| Error(Coin) | 1416.30 | 1086.32 | 1.30 |  |  |  |

**Logistic Regression for Study 2**

Identical to Study 1, logistic mixed-effects regression analyses were conducted for context and focal tower trials separately, with risky choice as a dependent variable (safe choice coded as 0 and risky choice coded as 1), context risk rate, learning format and coin as fixed effects, and participant as a random effect (S5 Table). The analyses were performed via the R software using the glmer function in lme4 package [1].

Consistent with the results from mixed-ANOVA analyses, context risk rate (*B* = -0.51, *z* = -10.42, *p* < .001) and coin (*B* = -0.03, *z* = 29.67, *p* < .001) were significant predictors predicting the changes in likelihood of risky choices in context tower trials. Similarly, in focal tower trials, context risk rate (*B* = 0.13*, z* = 2.91, *p* = .01) and coin (*B* = 0.03, *z* = 30.47, *p* < .001) were significant predictors predicting the changes in likelihood of risky choices. In both context and focal tower trials, learning format did not significantly predict changes in likelihood of risky choices.

**S10 Table. Logistic mixed regression on % risky choices for context and focal towers from Study 2**

|  | Predictor | Variance | *B* | *Std. error* | *z* | *p* |
| --- | --- | --- | --- | --- | --- | --- |
| Context Tower Trials | Random effects |  |  |  |  |  |
|  | RP (Intercept) | 1.16 |  | 1.08 |  |  |
|  | Fixed effects |  |  |  |  |  |
|  | (Intercept) |  | 0.13 | 0.05 | 2.74 | 0.01** |
|  | Learning Format |  | -0.05 | 0.05 | -0.95 | 0.340 |
|  | Context Risk Rate |  | -0.51 | 0.05 | -10.42 | <.001*** |
|  | Coin |  | 0.03 | 0.00 | 29.67 | <.001*** |
|  | Learning Format*Context Risk Rate |  | 0.05 | 0.05 | 0.99 | 0.323 |
|  | Learning Format*Coin |  | < 0.001 | 0.00 | -2.09 | 0.036* |
|  | Context Risk Rate*Coin |  | < 0.001 | 0.00 | -3.36 | <.001*** |
|  | Learning Format*Context Risk Rate*Coin |  | < 0.001 | 0.00 | -0.73 | 0.466 |
| Focal Tower Trials | Predictor | Variance | *B* | *Std. error* | *z* | *p* |
|  | Random effects |  |  |  |  |  |
|  | RP (Intercept) | 1.03 |  | 1.02 |  |  |
|  | Fixed effects |  |  |  |  |  |
|  | Intercept |  | 0.18 | 0.05 | 4.02 | <.001*** |
|  | Learning Format |  | 0.01 | 0.05 | 0.27 | 0.790 |
|  | Context Risk Rate |  | 0.13 | 0.05 | 2.91 | 0.01** |
|  | Coin |  | 0.03 | 0.00 | 30.47 | <.001*** |
|  | Learning Format*Context Risk Rate |  | -0.03 | 0.05 | -0.57 | 0.567 |
|  | Learning Format*Coin |  | < 0.001 | 0.00 | -3.27 | 0.01** |
|  | Context Risk Rate*Coin |  | < 0.001 | 0.00 | -1.31 | 0.191 |
|  | Learning Format*Context Risk Rate*Coin |  | < 0.001 | 0.00 | -0.05 | 0.962 |

*Note*. Asterisks indicate significance at p < 0.05 (*), p < 0.01 (**) and p < 0.001 (***)

**References**

1. Bates D, Mächler M, Bolker B, Walker S. Fitting linear mixed-effects models using lme4. arXiv Prepr arXiv14065823. 2014;
